# Autophagy Modulators Profoundly Alter the Astrocyte Cellular Proteome

**DOI:** 10.3390/cells9040805

**Published:** 2020-03-26

**Authors:** Affan Ali Sher, Ang Gao, Kevin M. Coombs

**Affiliations:** 1 Department of Medical Microbiology & Infectious Diseases, University of Manitoba, Winnipeg, MB R3E 0J9, Canada; sheraa3@myumanitoba.ca; 2 Manitoba Centre for Proteomics & Systems Biology, University of Manitoba, Winnipeg, MB R3E 3P4, Canada; Ang.Gao@umanitoba.ca; 3 Children’s Hospital Research Institute of Manitoba, Winnipeg, MB R3E 3P4, Canada

**Keywords:** cell responses, autophagy, BafilomycinA1, Rapamycin, aptamers, bioinformatics

## Abstract

Autophagy is a key cellular process that involves constituent degradation and recycling during cellular development and homeostasis. Autophagy also plays key roles in antimicrobial host defense and numerous pathogenic organisms have developed strategies to take advantage of and/or modulate cellular autophagy. Several pharmacologic compounds, such as BafilomycinA1, an autophagy inducer, and Rapamycin, an autophagy inhibitor, have been used to modulate autophagy, and their effects upon notable autophagy markers, such as LC3 protein lipidation and Sequestosome-1/p62 alterations are well defined. We sought to understand whether such autophagy modulators have a more global effect upon host cells and used a recently developed aptamer-based proteomic platform (SOMAscan^®^) to examine 1305 U-251 astrocytic cell proteins after the cells were treated with each compound. These analyses, and complementary cytokine array analyses of culture supernatants after drug treatment, revealed substantial perturbations in the U-251 astrocyte cellular proteome. Several proteins, including cathepsins, which have a role in autophagy, were differentially dysregulated by the two drugs as might be expected. Many proteins, not previously known to be involved in autophagy, were significantly dysregulated by the compounds, and several, including lactadherin and granulins, were up-regulated by both drugs. These data indicate that these two compounds, routinely used to help dissect cellular autophagy, have much more profound effects upon cellular proteins.

## 1. Introduction

There is growing appreciation for the roles of apoptosis and autophagy in numerous areas of scientific research, including their roles in normal development and differentiation, and their potential roles in viral infections and in tumor cell development and function. Autophagy is a complex cellular pathway that targets organelles, protein aggregates, and pathogen components for lysosomal degradation [1,2]. There are three general types of autophagic process: macroautophagy, microautophagy, and chaperone-mediated autophagy (reviewed in [3]). Macroautophagy involves the formation of a double membranous structure around the components, followed by fusion with lysosomes for the degradation of the trapped components [4]. Microautophagy is a non-selective degradative process involving the direct engulfment of cytoplasmic material by the lysosomal membrane [5]. Chaperone-mediated autophagy recognizes and specifically selects cytoplasmic components for degradation using Hsc70 chaperone protein [6,7,8]. This Hsc70 tags the substrate and brings it to the lysosomal membrane where it binds to LAMP-2A before being translocated inside the lumen after a series of LAMP-2A assembly and disassembly steps [8]. Inhibiting and enhancing these autophagy mechanisms can impact cells drastically.

Autophagy plays a significant role in healthy human astrocytes and also in glioblastoma cells [9]. Pathways like Akt signaling are linked with activating mTOR proteins which in turn not only modulate autophagy but also regulate cell-cycle progression; if something goes awry, cell size and growth can be affected [9]. Since such signaling pathways are so intricately involved in regulating a plethora of other core cellular processes, no single drug or treatment has been identified to date that can target them to prevent glioblastomas from developing and growing [10]. One study that examined the cytotoxic effects of rapamycin in U-251 cells in the presence of temozolomide showed an increase in the level of autophagic glioma cell death, highlighting the importance of autophagy modulation in cancer treatment [11]. This is promising, however numerous aspects based on the diverse impact this drug can have on the human brain prevent this treatment option from being 100% efficient. Thus, to achieve maximum pharmacologic effect while minimizing side effects, studies to examine the impact of rapamycin, and an agent such as bafilomycinA1 (BafA1) with opposing autophagic effect, need to be conducted. Drugs such as BafA1 play a major role in the removal of damaged/oncogenic proteins from inside cells, and since the induction of autophagy is important for maintaining cellular integrity which is associated with pro-survival signaling, cancers have been associated with mutations arising in these autophagy genes [12].

In this study, we focus on profiling the impact of two autophagy modulating drugs, namely BafA1, an autophagy inhibitor, and Rapamycin, an autophagy inducer, on the cellular proteome of U-251 astrocytoma cells. Both drugs are well known to affect brain cellular functions and degeneration. Rapamycin is able to cross the blood brain barrier [13,14], although it is still unclear whether BafA1 can cross this barrier [15]. We used the aptamer-based proteomic SOMAscan^®^ platform [16,17] to simultaneously profile 1305 intracellular U-251 proteins in cells treated with 2nM BafA1 or 200 nM rapamycin over three time points, compared to non-treated cells. We also assessed secreted extracellular cytokines to complement the intracellular proteomic screen, because autophagy modulation plays a critical role in the control of immune response to either pathogen invasion or during inflammation in the CNS [3,18,19,20,21,22]. Our results indicate that sub-toxic concentrations of both drugs impact not only proteins involved in autophagy, but also dramatically impact numerous other cellular networks, pathways, and biologic functions, including development, cell morphology, cell movement, cell death and survival, lipid metabolism, the cardiovascular system, neurological and immunological diseases, and protein synthesis. Analyses of the cytokines secreted by these in vitro U-251 cells after treatment with BafA1 or rapamycin reveal predicted in vivo compromise in the blood brain barrier (BBB) and in induction of leukocyte migration from the periphery into the CNS. The cytokines produced normally modulate synaptic control, learning, memory, and various autoimmune disease pathologies such as multiple sclerosis, autism, epilepsy, and Parkinson’s disease. With these data, informed decisions about the possible future use of these drugs for cancer treatment or other purposes can be made while minimizing negative unwanted effects.

## 2. Materials and Methods

### 2.1. Cells

Human U-251 glioblastoma astrocytoma (U-251 MG (formerly known as U-373 MG; European Collection of Authenticated Cell Cultures-ECACC 09063001)) were cultured in Dulbecco’s modified Eagle’s/Nutrient Mixture F-12 (DMEM/F-12) medium. Medium was supplemented with 10% Fetal Bovine Serum (FBS), non-essential amino acids, sodium pyruvate, and 2 mM *l*-glutamine. Cells were maintained at 37 °C in 5% CO_2_ and passaged every 2–3 days by trypsinization.

### 2.2. Pharmacologic Treatments

U-251 cells were grown to ~70% confluency for various experiments. Cells were treated with various concentrations of the autophagy inhibitor Bafilomycin A1 (BafA1) (Sigma Aldrich, St. Louis, MO, U.S.A.) or the autophagy inducer Rapamycin (Rapa) (Sigma Aldrich) for various periods of time.

### 2.3. Cell Viability

U-251 cells were treated with various concentrations of BafA1 or Rapa for 12, 24 and 48 h. Cell viability was measured using the WST-1 assay according to manufacturer’s instructions (Roche Diagnostics, Laval, QC, Canada), but adding 8 μL of WST-1 reagent instead of 10 μL into each well of a 96-well plate after above drug treatments. The cells were incubated for 1.5 h at 37 °C and then absorbances were measured at 440 nm and 610 nm. Absorbance at 610 nm was subtracted from that at 440 nm and the values of the treated samples were normalized with non-treated samples at each time point. A minimum of triplicate experiments were analyzed.

### 2.4. Protein Quantification

Non-treated and pharmacologically-treated U-251 samples were harvested at each of various time points (12, 24, and 48 h post-treatment—hpt) and washed 3× with > 50-volumes of ice-cold PBS to remove media and FBS. Washed cells were lysed with MPER^®^ (Pierce, Rockford, IL, USA) supplemented with 1× HALT^®^ Protease inhibitor (Pierce, Rockford, IL, USA). After cell lysis, lysates were centrifuged at 14,000× *g* for 15 min at 11 °C to remove insoluble cellular components and cell lysate protein quantities were determined by BCA^TM^ Protein Assay (Pierce, Rockford, IL, USA) and normalized to bovine serum albumin standards.

### 2.5. SOMAscan^®^ Analyses

Protein concentrations of BCA-determined cell lysates were adjusted to 200 ng/μL, and 70 µL of each sample submitted for SOMAscan^®^ analysis in-house on a SomaLogics^®^-licensed platform in the Manitoba Centre for Proteomics and Systems Biology as described [17,23,24]. Briefly, the SOMAscan assay is a novel proteomic tool that uses single-stranded DNA-based Slow Off-rate Modified Aptamer reagents (SOMAmers). These chemically modified nucleotides were selected based upon their capacity to bind to specific human proteins. The SOMAmers capture proteins in their native state, and, after a series of washing steps, are released and their quantities measured on DNA microarray chips. When the SOMAmers are used to probe a range of sample concentrations, they are capable of measuring femtomolar to micromolar quantities of proteins. We used the SOMAscan version 1.3 (SomaLogics, Denver, CO, U.S.A.), capable of simultaneously measuring 1305 distinct proteins in each of up to 88 samples [17]. Three biologic replicates of drug-treated samples collected at 12, 24, and 48 hpt, and of each time-matched non-drug-treated control (= 18 total samples) were simultaneously analyzed in a single SOMAscan 96-well plate. Results were reported in relative fluorescent units (RFU) for each sample, which are directly proportional to the amounts of target protein quantities in the initial samples, as confirmed by a standard curve generated for each protein-SOMAmer pair [17]. RFU differences between each non-drug-treated replicate and time-matched drug-treated replicate samples were analyzed as described below in statistical and bioinformatics analyses.

### 2.6. Western Blots

BCA-quantified protein samples were adjusted to load 20 μg of protein per gel lane. Samples were heated to 95 °C for 5 min and resolved by mini-12% sodium dodecyl sulfate polyacrylamide electrophoresis (SDS-PAGE) until the loading dye had just run off the bottom edge of the gel. The proteins were transferred to PVDF (Immobilon-P polyvinylidene difluoride membrane (Millipore, Etobicoke, ON, Canada)) for 2 h in ice-cold buffer, followed by overnight blocking of the membrane in 5% skim milk in 1× TBST. Primary antibodies were added to each blot at 1:1000 dilution in 1% milk/TBST overnight. Primary antibodies used were: rabbit anti-LC3β (Invitrogen-Thermo Fisher, Waltham, MA, U.S.A.) # L8919, rabbit anti-SQSTM1/p62 (Cell Signaling, Danvers, MA, USA, #5114), and mouse anti-β-actin (Cell Signaling #3700). After overnight binding, membranes were washed 3× with TBST and appropriate goat HRP-conjugated anti-rabbit (Cell Signaling # 7074) or anti-mouse (Cell Signaling # 7076) secondary antibodies were added for 1 h. The blots were washed 3 additional times with 1× TBST, developed with ECL western blotting peroxidase substrate for chemiluminescence and imaged with an enhanced chemiluminescence (ECL) detection machine (Amersham-Pharmacia Biotech, Buckinghamshire, U.K.); ImageJ was used to analyze each blot and each band in each blot was normalized to its respective actin control and to its time-matched non-drug-treated control band intensity.

### 2.7. Cytokine Arrays

BCA-quantified protein samples were adjusted to 2 mg/mL and provided to Eve Technologies (Calgary, AB, Canada;) for cytokine array analyses. Values were returned as relative fluorescence units and picogram and nanogram quantities assigned based upon parallel in-house standard curves.

### 2.8. Statistical and Bioinformatic Analyses

RFU values for each of the 1317 analytes (1305 human proteins and 12 internal controls) in each of 3 biologic replicates, each consisting of a non-treated sample and a time-matched drug-treated sample at 12, 24 and 48 h post-drug treatment were imported into Excel and converted to Log_2_ values. Fold-changes were determined for each of the six treated samples compared to their time-matched non-treated samples. The fold-changes were analyzed for significance by both Students *t*-test with 2 tails, and by Z-score analysis, as described [23,25]. Briefly, all fold-changes not deemed to be significant by *t*-test were examined by Z-score, expressing each value as its number of standard deviations away from the population mean. Each protein’s Z-score was considered significant if the average Z-score for that protein was > 1.96 σ or < −1.96 σ; and if the Z-score was < −1.96 σ in each of 2 or more replicates and was < −0.98 σ in no more than a single replicate, or if the Z-score was > 1.96 σ in each of ≥ 2 replicates and was > 0.98 σ in the remaining replicate. For increased stringency, we also applied a fold-change cut-off of 1.33-fold dysregulation (≥ 1.33-fold if up-regulated, or ≤ 0.750-fold if down-regulated) to those proteins considered significantly dysregulated. Fold changes and *p*-values were imported into DAVID, Network Analyst and Ingenuity Pathway Analysis (IPA^®^, Redwood City, CA, U.S.A.) for additional bioinformatics and pathway analyses. Western blot data were examined for significance by one-way ANOVA, using a significance cut-off of 0.05.

## 3. Results

### 3.1. Autophagy Markers Are Affected in U-251 Astrocytoma Cells by Bafilomycina1 and Rapamycin

**Figure 1:**
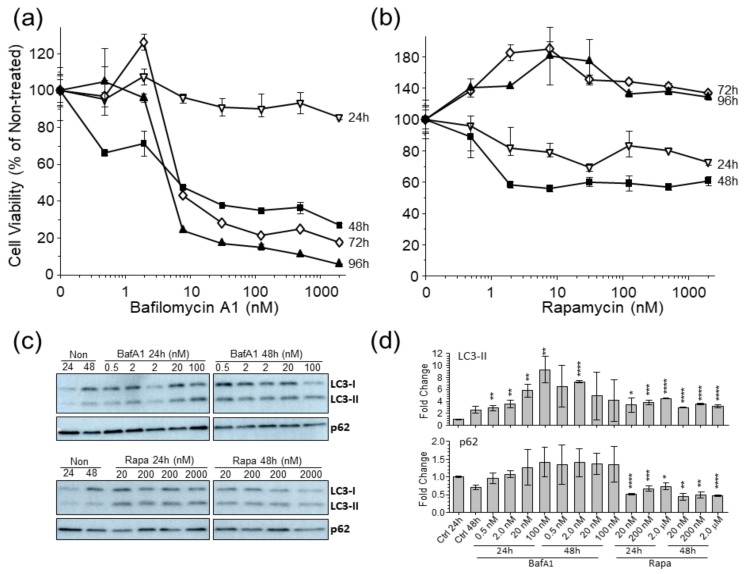
Effects of (**a**) BafilomycinA1 (BafA1) and (**b**) rapamycin (Rapa) on cell viability and (**c**,**d**) LC3-II conversion and p62 levels. (**a**,**b**) U-251 cells were treated with the compounds for indicated concentrations and periods of time, then cell viabilities determined by WST-1 assay. Results represent averages of four replicates; error bars are S.E.M. (**c**) Representative immunoblots of p62 and LC3 after treatment with indicated drugs for 24h. (**d**) Densitometric normalization to actin from three replicates. * *p* < 0.05; ** *p* < 0.01; *** *p* < 0.001; **** *p* < 0.0001.

To ensure that we treated our U-251 cells with sufficient concentrations of the autophagy modulating drugs BafA1 and Rapa, without inducing excessive cytotoxicity, we treated our cells with various concentrations of each drug for various periods of time and compared cell viability of the treated cells to non-treated cells. Cells treated with BafA1 for 24 h or less were mildly affected by BafA1 concentrations < 500 nM (Figure 1a). Cells treated for 48 h or longer with 2 nM BafA1 showed little cytotoxicity, but cytotoxicity increased dramatically at concentrations > 8 nM. Cells treated with 1000 nM Rapa for 72 h or less showed little cytotoxicity; cytotoxicity was apparent only at concentrations > 500 nM for 96 h of treatment (Figure 1b). We also probed drug-treated cells for alterations in the levels of autophagy markers LC3 and p62 (Figure 1c,d). BafA1 caused minor increases in p62 expression and significant increases in LC3 conversion (lipidation) at various tested times and concentrations. Rapa induced significantly more LC3 conversion, but significantly less p62 expression, compared to non-drug-treated. We selected doses of 2 nM BafA1 and 200 nM Rapa for subsequent experiments because of the anticipated need in later follow-up studies involving long-term treatment ± virus infection to incubate treatments for up to 48 h.

### 3.2. Bafa1 and Rapa Induce Dysregulation of Numerous U-251 Proteins

**Figure 2:**
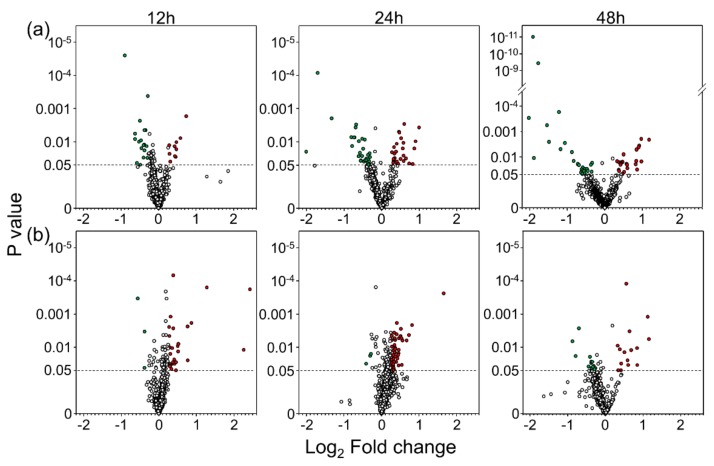
Volcano plots of (**a**) BafA1- and (**b**) Rapa-treated U-251 proteins after indicated times of treatment. Each protein measured by SOMAscan^®^ is represented by a circle. The dashed horizontal lines indicate *p*-value of 0.05 with circles above the lines being significantly dysregulated. Fold change cut-offs of +1.33 and -1.33 (± 0.415 Log_2_) are indicated with red and green filled circles, respectively. Values represent averages of three replicates.

We treated U-251 cells with 2 nM BafA1 or 200 nM Rapa for 12, 24 and 48 h, harvested the drug-treated cells and compared the cellular proteomes to time-matched non-drug-treated cells, using a SomaLogics^®^ version 1.3 platform. Each of 1305 human proteins was examined and relative quantities determined. Values for each of the drug-treated samples were normalized to the non-drug-treated samples from the same replicates and these comparative values were then analyzed. More than 300 proteins were significantly dysregulated by drug treatment (Table 1). Most of the dysregulated proteins were affected < 20%. Thus, we considered more stringent parameters and chose fold-change cut-offs of 1.333-fold (= down-regulated to 0.750 of non-drug-treated). Using these parameters, we identified 76 proteins that were significantly dysregulated by drug treatment (Table 1, Table 2 and Figure 2). Considerably more proteins were up-regulated by BafA1 treatment, whereas a slightly larger number of proteins were usually down-regulated by Rapa treatment (Table 1).

### 3.3. Bafa1 Generally Activates the U-251 Proteome and Rapa Generally Inhibits the U-251 Proteome

**Figure 3:**
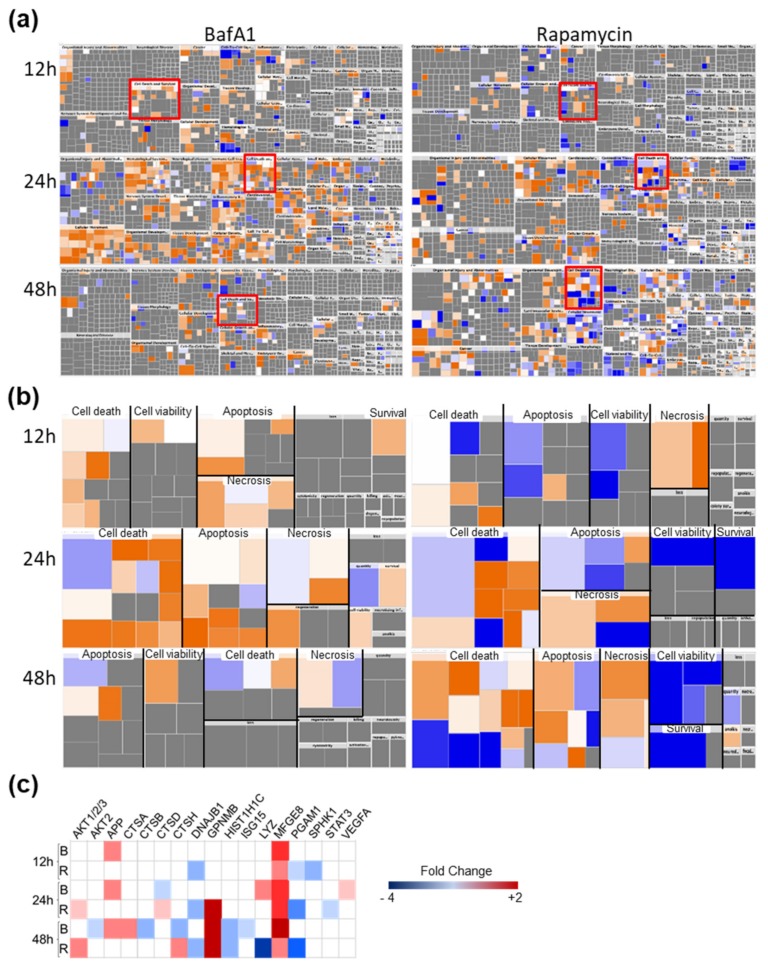
Global dysregulation of U-251 proteins induced by Bafilomycin (BafA1) and Rapamycin (Rapa) treatment. U-251 cells were treated with the drugs for the indicated amounts of time, then cellular proteomes probed by SOMAscan^®^ and compared to non-treated time-matched samples. (**a**) Global Ingenuity Pathway Analysis (IPA) default-determined alterations in “Diseases and Functions”. The “Cell Death and Survival” categories in (**a**) are enclosed in red boxes and expanded in (**b**). (**b**) Expanded Cell Death and Survival categories with each sub-category “Apoptosis”, “Cell death”, “Cell viability”, “Necrosis” and “Survival” separated. Each colored block within each sub-category refers to specific cellular nodes. Up-regulated (activated) nodes are indicated in orange; down-regulated (inhibited) by blue; not significantly regulated in grey. (**c**) Significantly dysregulated proteins associated with autophagy induced by BafA1 (B) or Rapamycin (R). Determinations based on three biological replicates.

We imported protein dysregulation levels and identifications into the Ingenuity Pathway Analysis (IPA) tool. For this, we expanded the dataset of dysregulated proteins to those significantly dysregulated > 1.25-fold to increase the number of analyzed molecules to >100. Both drugs induced significant alterations in the global U-251 proteome at multiple time points, with most dramatic effects occurring by 24 h treatment or later (Figure 3). As reflected by the numbers of dysregulated proteins induced by each treatment (Table 1, Table 2, Figure 3a), BafA1 demonstrated a moderately positive overall Z-score in many categories (orange = activation) and Rapa-treated cells showed overall negative Z-scores (blue; Figure 3a), particularly in the “cell death and survival” categories (Figure 3b). For example, the 24h BafA1 treatment showed many more activated cell death, apoptosis and necrosis nodes than inhibited nodes. There were approximately equivalent numbers of activated and inhibited cell death, apoptosis and necrosis nodes in the Rapa 24h datasets, but the cell viability and survival nodes were overall inhibited. By 48h Rapa treatment, there was a greater shift towards cell death, as represented by increased activation of cell death processes and concurrent inhibition of cell survival processes. Examination of specific proteins known to be involved in autophagy showed that MFGE8 was increased by both treatments, but several other proteins were differentially regulated (Figure 3c). APP was up-regulated only by BafA1 treatment, DNAJB1 was down-regulated only by Rapa treatment, and CTSD and CTSH were differentially regulated by the two agents, consistent with the differential inhibitory and enhancing activities, respectively, of these two drugs.

**Figure 4:**
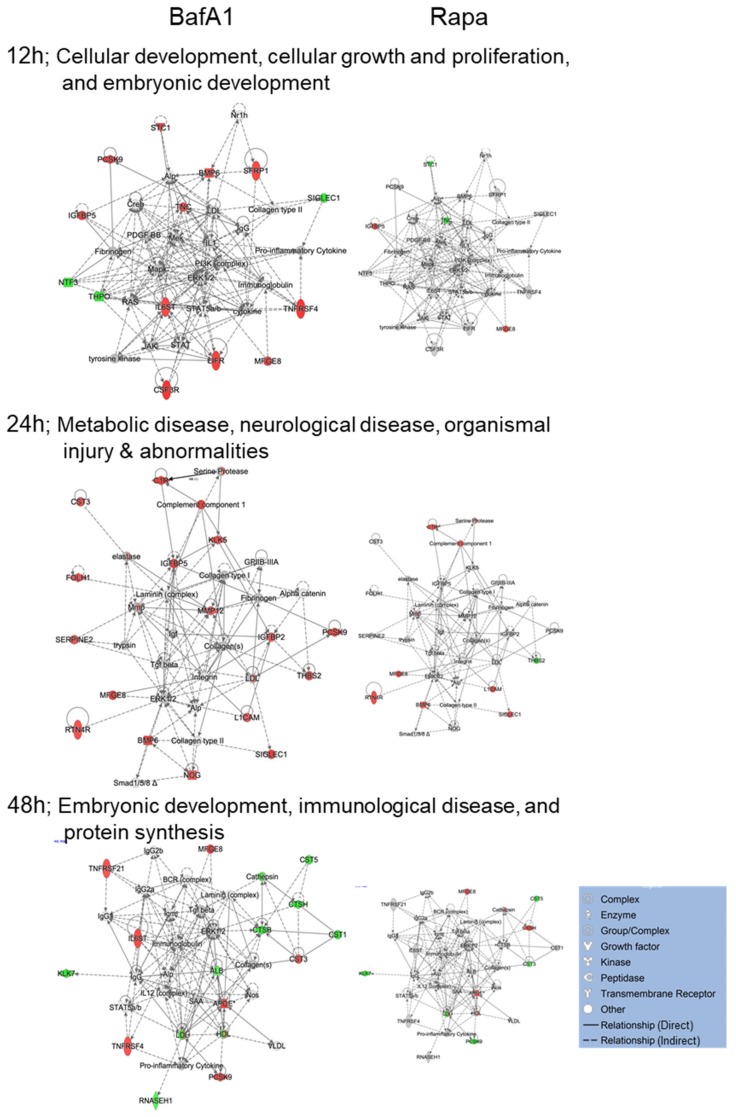
IPA-determined top cellular Networks affected by 2 nM BafilomycinA1 (BafA1). Top BafA1-affected network at each time (left), with 200 nM rapamycin data overlaid (right). The top rapamycin-affected networks are shown in Figure 5. Red proteins are significantly up-regulated; green are down-regulated; grey proteins were not significantly dysregulated; white proteins are part of the network but not covered by the SOMA panel. Protein types indicated in legend.

**Figure 5:**
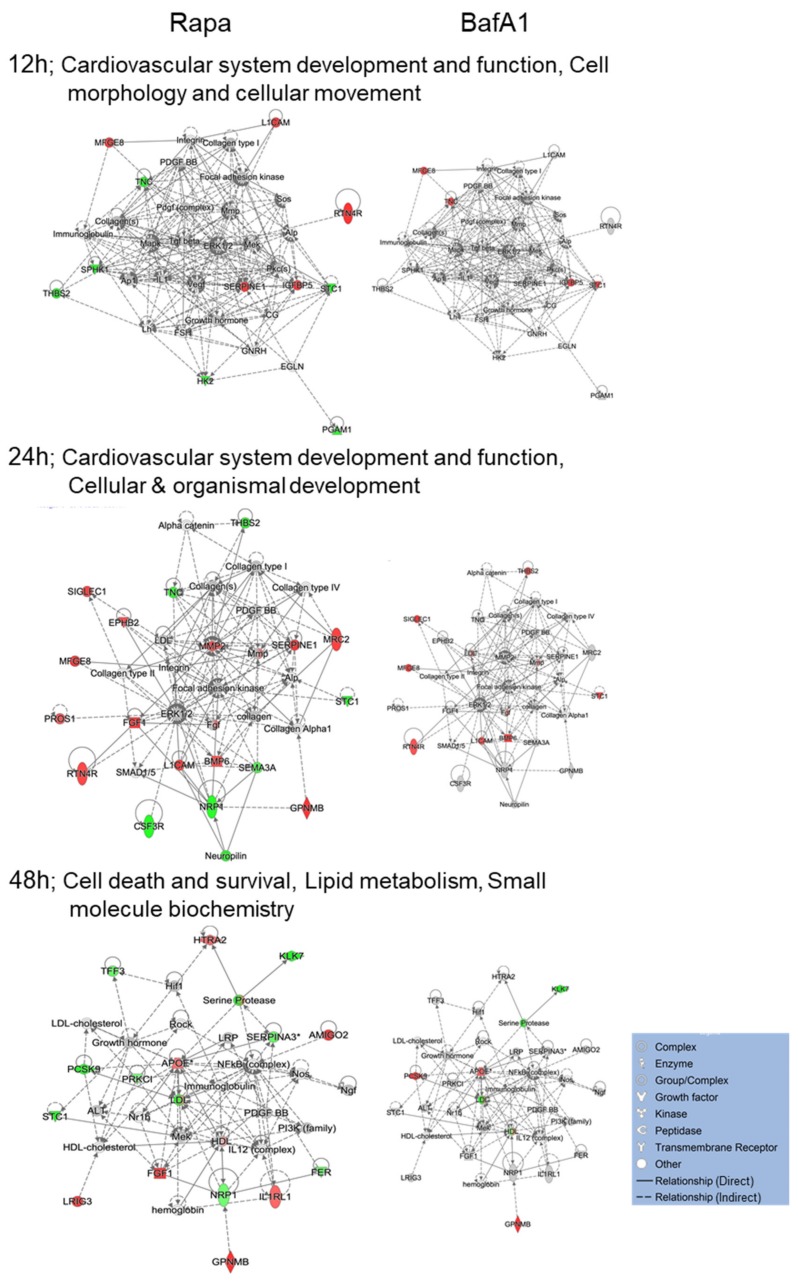
IPA-determined top cellular Networks affected by 200 nM rapamycin (Rapa). Top Rapa-affected network at each time (left), with 2 nM BafA1 data overlaid (right). The top BafA1-affected networks are shown in Figure 4. Red proteins are significantly up-regulated; green are down-regulated; grey proteins were not significantly dysregulated; white proteins are part of the network but not covered by the SOMA panel. Protein types indicated in legend.

IPA network analyses also revealed differences in how each drug affected common cellular networks. The highest scoring Networks affected by BafA1 treatment at different time points were Cellular development, growth and proliferation; Metabolic and neurological disease; and Embryonic development, immunological disease and protein synthesis (Figure 4). The highest scoring networks affected by rapamycin treatment at different time points were cardiovascular system development and function (at both 12 and 24 hpt), and later at 48 hpt, cell death and survival, lipid metabolism and small molecule biochemistry (Figure 5). Several proteins (i.e., MFGE8, SIGLEC1, L1CAM, BMP6, Fgf, KLK7, Serine protease, IGFBP5, RTN4R, C1R, and APOE) are similarly dysregulated by both treatments. However, consistent with these two drugs having differential biological effects, more proteins were differentially dysregulated by the two drugs. For example, STC1 and TNC were up-regulated by BafA1 at 12 h but down-regulated by Rapa, THBS2 was up-regulated by BafA1 at 24 h but down-regulated by Rapa, PCSK9 was up-regulated by BafA1 at 48h but down-regulated by Rapa, CTSH was up-regulated by Rapa at 48 h but down-regulated by BafA1, and numerous other proteins (i.e., CSF3R, FGF1, MMP2, EPHB2, PRKCI, NTF3, THPO, CST3, MMP12, RNASEH1, TNFRSF4, TNFRSF21, IL6ST and CTSB) were significantly dysregulated by one agent but not by the other.

### 3.4. Bafa1 and Rapamycin Differentially Affect U-251 Bio-Functions

**Figure 6:**
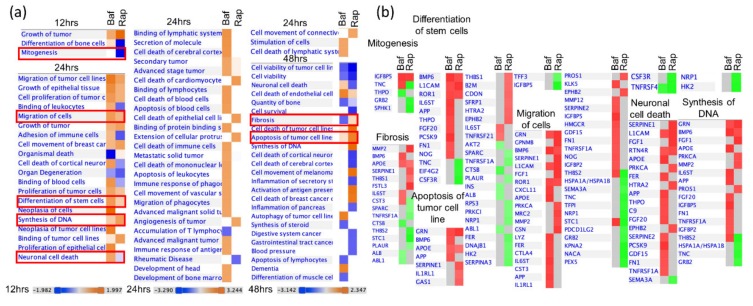
IPA-determined bio-functions altered by 2 nM BafA1 or 200 nM rapamycin. (**a**) Altered bio-functions with Z-Score > 1.96 (orange) or < −1.96 (blue) at each time point. (**b**) Some of the bio-functions (outlined in red in **a**) are shown in greater detail, with dysregulation of specific proteins indicated (red blocks indicate up-regulated; green indicates down-regulated).

Various bio-functions also were examined using the IPA^®^ default settings for these analyses (Figure 6). Bio-function activation is assumed for Z-scores > 1.96 σ, and bio-function inhibition is assumed for Z-scores < −1.96 σ. All these indicated bio-functions also had significant *p*-values. Most dysregulated functions occurred at 24 hpt. Although a few functions (i.e., stem cell differentiation, DNA synthesis, and cell migration) were bio-functions predicted to be similarly dysregulated by both drugs, many more bio-functions were dissimilarly dysregulated (i.e., fibrosis, apoptosis of tumor cells, binding of leukocytes, adhesion of immune cells, and neuronal cell death) (Figure 6).

**Figure 7:**
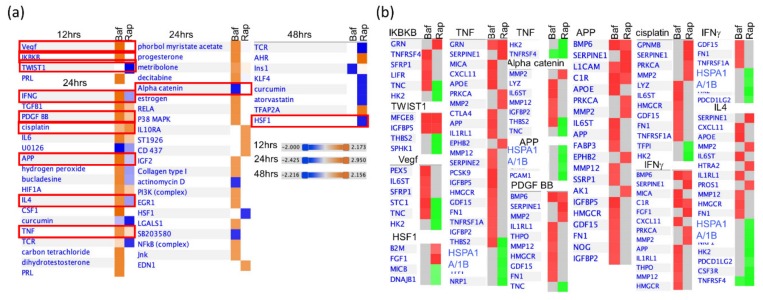
IPA-determined upstream molecules altered by 2 nM BafA1 or 200 nM rapamycin. (**a**) Predicted affected upstream molecules with Z-Score > 1.96 (orange) or < −1.96 (blue) at each time point. (**b**) Some of the upstream molecules (outlined in red in **a**) are shown in greater detail, with dysregulation of specific proteins indicated (red indicates up-regulated; green indicates down-regulated).

We then used IPA to examine predicted upstream molecules (Figure 7). Although a few molecules (i.e., TGFB1, PDGF BB, cisplatin, and TNF) were predicted to be similarly dysregulated by both drugs, many more molecules were predicted to be differentially affected (i.e., Vegf, IKBKB, APP, PDGF BB, TNF, IL4, and alpha catenin) (Figure 7). For example, TNC, which is involved in IKBKB, Vegf events was significantly up-regulated by BafA1 treatment, but down-regulated by Rapamycin treatment. Similarly, THBS2, involved in alpha catenin events, was significantly differently dysregulated.

### 3.5. Bafilomycin and Rapamycin Differentially Affect Levels of Cytokine Secretion

Several of the molecules probed by the SOMAscan^®^ array are cytokines and chemokines. To validate some of the SOMAscan-determined dysregulations, which were performed on cell lysates, and to complement the above analyses, we also examined secretion of numerous cytokines after U-251 treatment with the two drugs. Concentrated supernatants from cells treated with either compound, or not treated, for 16 h and 42 h, were submitted to Eve Technologies (Calgary, AB, Canada) for analysis. Many more cytokines were significantly up-regulated by both compounds than were down-regulated (Table 3). For example, TPO (thrombopoietin), Eotaxin, MCP-3 (monocyte chemotactic protein-3), and others were up-regulated by both compounds at both tested times. LIF (leukemia inhibitory factor) was down-regulated by both compounds at both tested times. Interleukin (IL)-1α was up-regulated by BafA1 at both times and down-regulated by Rapa at early times post-treatment.

## 4. Discussion

### 4.1. Implications of Bafa1/Rapa-Dysregulated Intracellular Astrocytic Proteins

The role of autophagy has been examined in a variety of scenarios, including cell proliferation and differentiation during embryogenesis, postnatal development, neuronal development and protein turnover during neurogenesis, and sregulation of cancer cell survival [26,27,28]. In addition, numerous ubiquitous cellular processes involving growth factor regulation and intracellular amino acid and energy status maintenance are also manipulated by either the inhibition or activation of autophagy [29]. This has led to further studies that have demonstrated potential activation of autophagy in G1 and S phases of the cell cycle via binding of p14^ARF^, an inhibitor of G1 progression, to BCL-XL, preventing Beclin1 from binding to it, to a relative inhibition of autophagy during mitosis [29]. Furthermore, senescence, an irreversible cell cycle arrest process, has also been linked directly to the role of autophagy [29]. Many of these broad cellular functions have not been clearly characterized in terms of the expression/activation profile of the underlying molecules involved. Our proteomic study used an approach to study > 1300 cellular proteins and how each of them might be affected by drugs known to modulate autophagy.

We chose BafilomycinA1 (BafA1) and rapamycin (Rapa), two agents commonly used to study autophagy. BafA1 functions as a V-ATPase inhibitor and Rapa activates autophagy by inhibiting mTOR [3,30,31]. The V-ATPase function is important because it activates lysosomal enzymes to allow the cells to degrade autophagic cargo for recycling and cell survival [30]. Other pharmacologic agents with similar effects (i.e., chloroquine, which has been used as an anti-malarial and anti-viral for many decades) could play similar roles. Although the two drugs, BafA1 and Rapa, have overall opposing effects on autophagy, numerous cellular targets were similarly dysregulated by both drugs (Figure 3, Figure 4, Figure 5, Figure 6 and Figure 7). These have important functions pertaining to brain development and function. For example, up-regulation in L1CAM, a member of the Immunoglobulin superfamily, is observed 24 h post BafA1/Rapa treatment; this specific target is regulated by fibronectin domains (which also are overexpressed in the presence of BafA1 treatment at 24 h (Table 2)). Disruption of FN results in not only the obstruction of protein trafficking but also a plethora of mental disorders such as mental retardation, limb spasticity, and hydrocephalus [32,33]. Other protein dysregulations included the up-regulation of MGF-E8, EPH-B2, and FGF1 and down-regulation of THBS2 and PCSK9 (Figure 2a). Among the top dysregulated networks in Rapa-treated U251 cells, interactions between a variety of molecular targets involved in cell development, function, survival, metabolism and morphology, and movement were highlighted (Figure 3a).

Two proteins, MFG-E8 and SPARC, are down-regulated in the presence of Rapa at 48 hpt (Table 2). These proteins have been associated with astrocyte-synapse and astrocyte-endothelial cell interactions and in the suppression of A1 reactive astrocytes via the up-regulation of PI3K-Akt and the down-regulation of nuclear factor-κB pathways [34,35]. MFG-E8 also has a protective role against Alzheimer’s Disease by upregulating the induction of neurogenesis and neural stem cell proliferation [36]. In addition to MFG-E8 up-regulation, which cues dysregulation in synaptic functions, one of the receptor tyrosine kinases, EphB2, that binds to ephrin-B ligand stimulating NF-κB pathway also causes cytokine expression via the MAPK pathway [37]. EphB2 is not only crucial for synapse functioning but also plays a role in astrocytic activation during pro-inflammatory response and neuronal excitotoxicity [37,38]. FGF1 is another dysregulated protein that impacts astrocytic activation [39]. All these dysregulations converge towards the core concept that use of BafA1 and Rapa can impact crucial aspects of brain physiology and morphology.

In examining other astrocytic targets, THBS2 is a target that is down-regulated by Rapa but up-regulated by BafA1 (Table 2). Since THBS2 is part of the astrocytic excretory product, its lower intracellular expression indicates a potential impact the drugs are most likely to have on synaptogenesis, neurite outgrowth, cell attachment, migration and proliferation, as has also been indicated by IPA analyses (Figure 3) [40,41]. Another dysregulated protein, PCSK9, has a major role in cholesterol homeostasis via the degradation of LDL receptor [42]. This is known to happen during brain development and after ischemic stroke in mice [42]. In addition, deviations in PCSK9 levels have been correlated with other Alzheimer’s disease biomarkers during the pre-onset stage of the disease [43].

Dysregulation of proteins like HK2, which are a crucial part of the glucose metabolic pathways, points towards alterations in the astrocytic metabolism upon treatment with these drugs, which is crucial when determining the use of these drugs for clinical purposes. Since we are using the U-251 glioblastoma cell line as a model for astrocytes, the oncological effects of down-regulation of CSF3R, (Granulocyte-colony stimulating factor receptor), pave the way for repurposing these drugs towards cancer treatment as down-regulation of G-CSF/G-CSFR signalling is known to inhibit growth and metastasis of glioma cells [44].

In-depth analyses of the most significantly activated/inhibited activity patterns using the IPA software indicated that numerous biological functions overlap with the dysregulated functions described in the previous paragraph. Proteins like protein kinase C, MMP2 and BMP6 have important implications in cell cycle progression, tumorigenesis and metastasis; thus, these results help in understanding and connecting the significance of BafA1/Rapa treatment on astrocyte-derived glioblastoma cells as a potential crucial role in cancer research [45,46]. Prior research has elucidated the role of autophagy in mammalian development; thus, this study expands prior work by examining a vast variety of mammalian targets that are modulated when astrocytic cells are treated with BafA1 or Rapa (Figure 3b) [27]. Many of our identified targets, such as MMP2, are involved in functions like fibrosis and cell migration (Figure 3b). THBS-1 and -2 are another pair of targets with dysregulated expression involved in fibrosis, apoptosis of tumor cells and synthesis of DNA (Figure 3b). Many of these proteins, like FGF1, EPHB2, PCSK9, and SPARC, have been mentioned above in functions like maintaining astrocytic/neuronal connections, and a variety of diseases associated with dysregulation in either loss of development of these connections. Thus, linking these protein targets to brain developmental effects induced by BafA1 or Rapa seems plausible. Other proteins, like BMPs, TNC, PKC, and CST3 could potentially and indirectly aid either of the two drugs in having impacts on the cellular processes shown in Figure 3a by their roles in astrocytic maturation and mitogenesis, migration, tumor invasion, and controlling DNA checkpoints required for maintaining genomic integrity [45,47,48,49].

IPA also was used to generate a holistic pattern of cell-wide activation/inhibition patterns for bio-functions (Figure 6) and upstream molecules (Figure 7). Some of the proteins were similarly affected by the two drugs whereas more proteins were dissimilarly affected. (Figure 6b, Figure 7b). Among the upstream molecules with dysregulated activation/inhibition patterns, VEGF, IKBKB, IFNG, PDGF BB, APP, TNF and HSF1 are a few that are crucial in astrocytic functions as well as play a role in glioblastoma functioning and morphology (Figure 7a). VEGF has been shown in mice to be critical in the maintenance of the blood brain barrier permeability with the implications of a decrease of its function being manifested in multiple sclerosis as well as astrocytic differentiation [50,51]. IKBKB, IFNG and PDGF BB are other proteins that have been shown to be involved in reactive gliosis, inflammation, cellular plasticity, neurogenesis, protection against autoimmune demyelination, and activation of astrocytes, especially during certain viral infections such as HIV-1 [52,53,54,55,56]. IFNG also reduces autoimmune encephalomyelitis in animal models upon its silencing [57].

### 4.2. Implications of Bafa1/Rapa-Dysregulated Secreted Cytokines

Owing to the presence of specialized endothelial cells in the CNS parenchyma that form the blood–brain barrier and the specialized epithelial cells in choroid plexus that form part of the blood cerebrospinal fluid barrier, the CNS has long been considered an immune-privileged part of the body, but no more [58]. Numerous pieces of evidence suggest that immune responses occurring in the CNS are not only facilitated by internal microglia, but also by the penetration of immune cells like CD4^+^ activated T helper cells, CD8^+^ cells, B cells, monocytes, and dendritic cells from the periphery. The implications of this are numerous, including immunological surveillance, modulation during viral/bacterial CNS infections, and autoimmune disorders like multiple sclerosis (MS) [58,59,60]. Cytokines play crucial roles in these immune responses. They are secreted by not only the immune cells but also microglia, astrocytes and neurons; they include a variety of signalling molecules like interleukins, chemokines, interferons, tumor necrosis factors (TNF), and transforming growth factors (TGF) [61]. Since these molecules are critical modulators of inflammatory responses in the CNS innate immune system, their levels need to be tightly regulated [62]. We therefore included complementary cytokine analyses to determine potential BafA1/Rapa impacts on the astrocytic secretome that could potentially lead towards either aggravation or alleviation of a variety of mental conditions mentioned above [60,62,63]. Many more cytokines were over-expressed than were under-expressed. These included a plethora of proinflammatory ones (e.g., IL1A, IL1B, IL6, TNFA, IFNG, TGFB, GM-CSF, IL12, IL17, IL18, CCL2, CCL5, and CX3CL1), some anti-inflammatory ones (IL4, IL6, IL10 and IL13), and some dual functioning, such as IL6 [64,65].

BafA1 and Rapa both induced up-regulation in the expression of numerous cytokines (e.g., TNFA, CXCL10, IL1B, IL1A, CCL2 and G-CSF (Table 3)) that result in not only the recruitment and infiltration of monocytes, CD4^+^, and CD8^+^ T cells to the CNS, but also promote the induction of neuroinflammatory reactive astrocytes and activation of microglia [58,60,66,67,68]. Since astrocytes have a role in phagocytosis of myelin debris and reactive neuroinflammatory astrocytes are unable to do so, observed alterations point towards a potential loss of axon growth in the drug-treated CNS [68]. Even GM-CSF, a crucial molecule which is involved in activation and differentiation of monocytes to antigen presenting cells (APCs), allowing them to interact with CD4^+^ cells, gets over-expressed 40h post BafA1/Rapa treatment despite being under-expressed at 16h (Table 2) [66]. The infiltration of immune cells into the CNS is also made possible by the upregulation of IL1B, CCL2 and TNFA that cause internalization of the tight junction proteins, which eventually compromise the blood brain barrier [66]. Another up-regulated cytokine, CXCL5, also results in neuroinflammation and BBB damage via its potential interaction with CXCR2 [69]. CXCL5 is unique in that it further enhances infiltration of neutrophils during CNS infections with HIV-1 and West Nile virus, leading to viral-induced encephalitis and related CNS pathologies [70]. The CNS susceptibility to WNV-mediated encephalitis [71] can also be a function of the up-regulation of CCL2 response we observe in both BafA1- and Rapa-treated U-251 cells. The almost 45-fold up-regulation of CCL27 by BafA1 is also consistent with increased lymphocytic trafficking, especially autoreactive T cells, into the CNS, which may lead to an even higher inflammatory brain response [72,73].

Many BafA1- or Rapa-induced up-regulated pro-inflammatory cytokines (e.g., IL-1B, TNFs, IL10, IL12, and IL13), correspond to the presence of CNS pathologies like cognitive impairment observed in patients with meningitis [60]. Other markers (e.g., IL6, TNFA, IL2 and IL4) were also up-regulated and are increased in the CNS of patients with viral encephalitis and autoimmune epilepsy [66]. IL1A and IL1B are another pair of cytokines that are highly up-regulated, especially in BafA1-treated cells (Table 3). Since IL-1 is expressed in the brain under normal conditions and is responsible for activation of neurons, regulation of sleep, synaptic plasticity, and adult neurogenesis, such an extreme up-regulation is likely to result in numerous human pathologies [74]. IL-1 dysregulation correlates in patients with rheumatoid arthritis [74], which implies potential aspects that need to be addressed before this drug makes its way past clinical trials. TNFα, IL2, and IL6 are another set of dysregulated cytokines that play crucial role in synaptic plasticity, homeostatic control, glutamate mediated cytotoxicity, potentiation, sleep, and communication between the immune and endocrine systems [62,64,75]. Further analysis of TNFα is crucial because this cytokine holds a central position in relation to a plethora of CNS conditions, including CNS-TB, autism and multiple sclerosis [64,76,77]. MS patients also demonstrate up-regulation in many of these cytokines in their CSF and serum, all of which are found to be up-regulated upon treatment with BafA1 and some with Rapa (Table 3) [73]. Finally, BafA1 and Rapa also result in massive up-regulation of IL9 (Table 3). IL9 receptors are present on astrocytes, oligodendrocytes and microglia, and IL9 dysregulation has been reported in CNS pathologies such as autoimmune encephalitis, oligodendrocyte progenitor cell proliferation and differentiation. Thus, such drug treatment that may mimic these CNS pathologies in patients being administered these drugs, warrants extreme caution and further investigation [77].

Down-regulation of specific cytokines also points towards the dysregulation of CNS immune responses. For example, PDBF-BB, down-regulated by both BafA1 and Rapa (Table 3), may lead to up-regulation of TNFA since PDGF-BB signaling is known to decrease TNFα secretion in the brain [78]. Another down regulated cytokine, CXCL12, also modulates leukocyte migration into the CNS parenchyma via the perivascular spaces, but the exact mechanism is still unknown and further studies targeting its upstream and downstream molecules are warranted to unravel the entire mechanism [59,63].

Utilization of the SOMAscan platform allowed us to examine relative quantities of a large number of specific proteins affected by numerous treatments. However, this platform is limited to the 1305 specific proteins designed to be recognized by the specific SOMAmers and alternative techniques, such as quantitative mass spectrometry, would be needed to more fully probe drug-induced cellular proteomic alterations. Furthermore, these types of analyses should be extended to in vivo animal models before benefits can be fully realized.

## 5. Conclusions

In conclusion, these dysregulated protein markers within cells and the cytokine secretome, cellular networks, functions and upstream molecules highlight the numerous ways these two autophagy modulating drugs, bafilomycinA1 (BafA1) and rapamycin (Rapa) can alter brain cellular proteins and impact neural circuits and functions. Most of these proteins and their functions converge on modulating autophagy. BafA1 and Rapa not only control cell recycling and degradation machinery, but also impact broader cellular functions, such as cell development, survival, metabolism, tumorigenesis, cell cycle progression and migration. In addition, numerous astrocyte-specific functions are impacted by the drugs, including synaptic plasticity, homeostatic control, glutamate-mediated cytotoxicity, and potentiation. Alterations in these can lead to numerous medical conditions, including CNS inflammation, autoimmune disorders, and neurodegeneration. Thus, additional careful studies of these aspects are warranted if they are to be used in drug therapies or clinical trials. In addition, since rapamycin is currently FDA approved and is used for the treatment of a variety of cancers and lymphomas, knowledge of the capabilities of this drug in modulating cellular development, migration, and proliferation in human astrocytes (one of the most abundant brain cells) is crucial in order to not only avoid potential harmful side effects, but also to potentially repurpose this drug for use in the treatment of other types of cancers. However, BafilomycinA1 is not FDA approved yet and this proteomic study provides more information on the potential effects of this drug.

## Figures and Tables

**Table 1:** Numbers of significantly dysregulated proteins at indicated cut-offs.

Number that Are Significant	Total Unique	BafilomycinA1			Rapamycin		
		12 h	24 h	48 h	12 h	24 h	48 h
and fold-change > 1.000	309	73	11	139	28	16	25
and fold-change < 1.000		15	51	14	38	22	27
and fold-change > 1.150	256	40	76	15	11	28	25
and fold-change < 0.870		6	6	18	27	35	26
and fold-change > 1.250	118	20	30	15	7	24	24
and fold-change < 0.800		3	1	12	15	30	26
and fold-change > 1.333	76	9	16	11	7	15	20
and fold-change < 0.750		1	0	4	8	19	23
and fold-change > 1.500	44	4	4	8	1	8	11
and fold-change < 0.667		0	0	3	3	10	16
and fold-change > 2.000	14	0	0	2	0	0	1
and fold-change < 0.500		0	0	0	0	2	9

Significance determined by *t*-test and Z-score as described in Materials & Methods. The 76 specific proteins significantly dysregulated > 1.33-fold are listed in Table 2.

**Table 2:** U-251 proteins significantly dysregulated by Bafilomycin A1 or Rapamycin.

EntrezGene Symbol	Protein	Bafilomycin A1			Rapamycin		
		12 h	24 h	48 h	12 h	24 h	48 h
Up-regulated by drug treatment *							
PCSK9	Proprotein convertase subtilisin/kexin type 9	1.83	3.15	2.22	0.92	0.93	0.25
STC1	Stanniocalcin-1	1.70	1.46	1.28	0.67	0.31	0.34
MFGE8	Lactadherin	1.70	1.76	1.79	1.50	1.56	1.39
TNFRSF4	Tumor necrosis factor receptor superfamily member 4	1.43	0.75	1.27	1.01	0.58	1.03
PEX5	Peroxisomal targeting signal 1 receptor	1.43	0.81	1.02	1.08	0.70	0.95
APP	Amyloid beta A4 protein	1.43	1.41	1.50	1.08	1.06	1.09
CSF3R	Granulocyte colony-stimulating factor receptor	1.42	0.82	1.12	1.04	0.67	1.03
FTH1 FTL	Ferritin	1.37	1.46	2.17	1.21	1.37	1.43
IGFBP5	Insulin-like growth factor-binding protein 5	1.37	1.60	0.99	1.34	1.00	0.43
BMP6	Bone morphogenetic protein 6	1.30	1.66	1.56	1.22	1.30	1.21
LAMA1 LAMB1 LAMC1	Laminin	1.31	1.59	1.19	1.14	1.02	0.56
APOE	Apolipoprotein E (isoform E4)	1.12	1.47	1.42	1.04	1.14	1.25
IL6ST	Interleukin-6 receptor subunit beta	1.28	1.45	1.33	1.06	1.03	0.98
CXCL11	C-X-C motif chemokine 11	0.89	1.43	0.92	0.92	1.23	0.76
LYZ	Lysozyme C	0.98	1.42	0.32	0.95	1.12	0.27
CST3	Cystatin-C	1.33	1.38	1.33	1.17	1.09	0.77
CTLA4	Cytotoxic T-lymphocyte protein 4	0.94	1.37	0.91	0.84	1.10	1.07
THBS2	Thrombospondin-2	1.21	1.37	1.08	0.73	0.65	0.48
KLK5	Kallikrein-5	0.93	1.37	0.80	0.97	1.08	0.65
ROR1	Tyrosine-protein kinase transmembrane receptor ROR1	1.03	1.36	0.96	1.02	1.32	1.09
FGF20	Fibroblast growth factor 20	1.08	1.34	1.03	0.98	1.05	0.92
GPNMB	Transmembrane glycoprotein NMB	1.08	1.18	1.79	1.16	1.87	1.82
GRN	Granulins	1.17	1.31	1.61	1.67	1.99	1.87
IGFBP2	Insulin-like growth factor-binding protein 2	1.24	1.27	1.52	0.89	0.74	0.54
CTSA	Lysosomal protective protein	1.02	0.97	1.47	1.19	1.22	1.27
PSMD7	26S proteasome non-ATPase regulatory subunit 7	1.04	1.14	1.00	1.39	1.42	1.06
AMIGO2	Amphoterin-induced protein 2	1.29	1.01	1.23	1.39	1.14	1.96
L1CAM	Neural cell adhesion molecule L1	1.09	1.32	1.07	1.37	1.50	0.79
RTN4R	Reticulon-4 receptor	1.19	1.27	1.12	1.35	1.34	1.01
C4A C4B	Complement C4	0.89	1.48	0.86	0.98	1.83	1.33
FSTL3	Follistatin-related protein 3	1.01	1.00	0.94	1.17	1.78	1.89
SERPINE1	Plasminogen activator inhibitor 1	1.19	1.26	1.01	1.31	1.66	1.52
FGF1	Fibroblast growth factor 1	0.96	1.18	0.85	1.00	1.52	1.77
LRIG3	Leucine-rich repeats and immunoglobulin-like domains protein 3	1.21	1.20	1.08	1.17	1.50	2.26
ICAM5	Intercellular adhesion molecule 5	1.06	1.25	1.16	1.15	1.49	1.25
ACY1	Aminoacylase-1	0.96	1.02	0.85	1.23	1.48	1.56
GSN	Gelsolin	1.03	1.12	1.01	1.15	1.43	1.11
MRC2	C-type mannose receptor 2	1.01	1.18	1.11	1.14	1.38	1.35
HTRA2	Serine protease HTRA2, mitochondrial	1.09	1.12	1.00	1.20	1.36	1.35
IDUA	Alpha-L-iduronidase	1.02	1.06	0.82	1.06	1.26	1.97
MMP2	72 kDa type IV collagenase	1.13	1.23	1.23	1.12	1.32	1.80
EFNA2	Ephrin-A2	1.14	1.06	1.03	1.14	1.22	1.50
GAS1	Growth arrest-specific protein 1	1.10	1.09	1.01	1.10	1.20	1.41
EPHB2	Ephrin type-B receptor 2	1.08	1.04	0.96	1.22	1.31	1.39
AKT1 AKT2 AKT3	RAC-alpha/beta/gamma serine/threonine-protein kinase	0.94	1.02	0.92	1.13	1.24	1.35
PROS1	Vitamin K-dependent protein S	0.97	1.19	0.94	1.09	1.27	1.34
Down-regulated by drug treatment *							
SIGLEC1	Sialoadhesin	0.68	1.56	1.01	0.87	1.39	0.92
CTSB	Cathepsin B	0.90	0.89	0.55	1.12	1.23	1.12
CTSH	Cathepsin H	0.92	0.84	0.58	1.08	1.22	1.51
HIST1H1C	Histone H1.2	1.02	1.01	0.61	1.05	0.96	0.62
CKB	Creatine kinase B-type	0.96	0.91	0.73	0.93	0.78	0.83
NACA	Nascent polypeptide-associated complex subunit alpha	1.02	0.98	0.75	0.54	0.62	0.44
CDC2 CCNB1	Cyclin-dependent kinase 1:G2/mitotic-specific cyclin-B1 complex	0.92	0.94	0.81	0.65	0.60	1.05
PFDN5	Prefoldin subunit 5	0.91	0.97	0.93	0.65	0.68	1.02
SPHK1	Sphingosine kinase 1	0.94	0.98	0.85	0.70	0.75	0.77
EIF4G2	Eukaryotic translation initiation factor 4 gamma 2	0.94	1.04	0.92	0.71	0.71	0.87
DNAJB1	DnaJ homolog subfamily B member 1	1.07	1.04	0.79	0.71	0.62	0.66
TNC	Tenascin	1.30	1.21	0.95	0.72	0.70	1.01
PGAM1	Phosphoglycerate mutase 1	0.95	0.89	0.84	0.77	0.40	0.36
CDK2 CCNA2	Cyclin-dependent kinase 2:Cyclin-A2 complex	0.92	0.83	0.98	0.68	0.57	1.79
HK2	Hexokinase-2	1.04	1.04	0.81	0.76	0.61	0.64
KPNA2	Importin subunit alpha-1	1.00	0.95	0.80	0.85	0.66	0.87
NRP1	Neuropilin-1	1.13	1.13	0.86	0.88	0.68	0.68
TFPI	Tissue factor pathway inhibitor	1.11	1.11	0.93	0.88	0.72	0.77
HSPA1A	Heat shock 70 kDa protein 1A	1.14	1.19	0.98	0.80	0.73	0.77
CFI	Complement factor I	1.08	1.11	0.86	0.78	0.73	0.76
TGM3	Protein-glutamine gamma-glutamyltransferase E	0.85	1.33	0.50	0.83	1.05	0.27
KLK7	Kallikrein-7	0.97	1.18	0.37	0.99	0.86	0.29
SERPINA3	Alpha-1-antichymotrypsin	0.98	1.31	0.84	1.05	1.23	0.61
ANG	Angiogenin	1.09	1.29	0.92	1.13	0.97	0.65
MICB	MHC class I polypeptide-related sequence B	1.11	1.14	0.94	0.96	0.81	0.67
TNFRSF1A	Tumor necrosis factor receptor superfamily member 1A	1.22	1.26	1.01	1.04	0.90	0.67
PES1	Pescadillo homolog	1.02	0.99	0.69	0.79	0.75	0.69
GFRA1	GDNF family receptor alpha-1	1.12	1.03	0.90	0.93	0.75	0.70
RPS3	40S ribosomal protein S3	0.98	1.04	0.98	1.02	0.81	0.72
PRKCI	Protein kinase C iota type	1.00	1.08	0.88	0.86	0.82	0.73

* Values represent protein fold-changes compared to non-treated. Fold-changes with significance < 0.05 are indicated in grey-shaded cells, with proteins significantly up-regulated ≥ 1.33-fold indicated in red, and proteins significantly down-regulated to ≤ 0.750-fold indicated in green. Proteins sorted first by up-regulation and from left-most column to right-most; then sorted by down-regulation from left to right.

**Table 3:** Secreted U-251 cytokines dysregulated by BafilomycinA1 or rapamycin.

	BafilomycinA1/Mock				Rapamycin/Mock			
	16 h		40 h		16 h		40 h	
	Baf/Untr *	*p*-Value	Baf/Untr	*p*-Value	Rap/Untr	*p*-Value	Rap/Untr	*p*-Value
*Up-Regulated Proteins*								
IL-1a	738	0.008	9714	0.001	0.40	0.071	1.47	0.113
TPO	7.72	1.8∙10^−4^	5.34	0.014	3.85	0.001	9.03	4.6∙10^−4^
Eotaxin	7.25	0.022	2.50	0.039	4.89	2.4∙10^−4^	2.95	0.002
MIP-1a	5.54	0.026	2.78	0.147	5.40	0.005	4.50	0.045
MCP-4	5.39	0.002	5.62	0.001	5.30	0.002	5.90	0.001
IL-12P70	4.34	0.003	11.35	0.036	7.56	0.078	8.92	0.085
MCP-3	4.18	0.001	7.29	7.6∙10^−5^	3.00	0.001	3.60	0.007
IFNa2	4.05	0.030	1.38	0.480	2.60	0.083	3.49	0.027
Fractalkine	3.36	0.001	0.88	0.155	1.98	0.005	2.36	0.013
IL-1RA	3.18	0.017	2.22	0.090	2.79	0.012	4.86	0.260
IL-13	2.95	0.011	1.16	0.465	1.94	0.075	3.78	0.003
IL-8	2.36	0.008	7.21	0.019	0.81	0.0503	1.89	0.001
IL-5	2.32	0.003	1.80	0.009	1.78	0.010	3.12	0.027
MCP-2	2.31	4.3∙10^−4^	3.99	0.001	2.83	0.026	5.03	0.001
BCA-1	2.27	0.010	1.04	0.447	2.26	0.001	2.51	9.9∙10^−5^
MCP-1	2.24	0.023	1.70	0.039	1.45	0.029	3.20	0.001
IL-3	2.22	0.037	1.19	0.119	0.96	0.845	2.69	0.340
IL-6	2.19	0.001	1.60	0.012	1.71	0.027	3.17	0.005
GRO alpha	2.03	0.002	6.70	1.6∙10^−4^	1.17	0.080	1.85	0.004
IL−9	44.31	0.195	47.07	8.9∙10^−5^	65.08	0.018	23.63	0.006
CTACK	30.10	0.102	45.12	0.002	31.66	0.180	21.95	0.303
TNFa	1.76	0.005	12.10	0.001	0.69	0.029	2.80	0.003
IL-1B	4.56	0.203	6.36	0.043	1.69	0.137	5.01	0.024
G-CSF	1.53	0.038	5.48	5.9∙10^−5^	5.13	0.001	3.66	0.003
IL-4	1.73	0.066	5.43	0.001	2.39	0.052	1.66	0.422
Eotaxin-2	1.30	0.026	3.96	0.002	0.97	0.561	1.83	0.004
ENA-78	2.88	0.180	2.94	0.020	1.38	0.605	6.15	0.004
IL-2	3.39	0.058	2.78	0.005	2.29	0.089	2.79	0.035
IP-10	0.73	0.0497	2.16	0.009	0.69	0.033	1.84	0.006
TNFB	2.66	0.180	1.88	0.190	2.16	0.334	7.80	0.037
PDGF-AA	1.96	0.008	1.50	0.015	1.42	0.021	3.17	0.006
EGF	1.74	0.005	1.30	0.023	0.92	0.238	2.79	0.005
IL-10	1.56	0.118	1.37	0.594	0.88	0.742	2.68	0.038
IL-28A	2.19	0.093	1.97	0.316	2.04	0.064	2.68	0.017
IL-12P40	1.31	0.061	2.49	0.113	1.67	0.127	2.33	0.043
IL-23	1.95	0.297	0.78	0.309	1.20	0.747	2.30	0.012
FGF-2	0.52	2.2∙10^−6^	1.03	0.006	0.93	0.003	2.26	4.9∙10^−4^
IL-7	1.00	0.945	1.23	0.026	0.62	0.011	2.26	0.012
Flt-3L	1.00	0.958	1.33	0.017	0.73	0.011	2.22	4.0∙10^−4^
*Down-Regulated Proteins*								
LIF	0.04	0.001	0.38	0.002	0.05	0.001	0.13	0.001
PDGF-BB	0.04	1.2∙10^−4^	0.33	3.0∙10^−4^	0.04	1.2∙10^−4^	1.23	0.003
TGF-a	0.16	0.004	0.46	0.010	0.17	0.003	1.42	0.025
GM-CSF	0.20	2.6∙10^−4^	6.34	0.005	0.07	2.0∙10^−4^	2.45	0.006
IL-18	0.30	0.001	0.39	3.8∙10^−4^	0.42	0.001	4.48	4.0∙10^−4^
Eotaxin-3	0.35	0.033	0.76	0.197	0.26	0.025	1.86	0.020
SDF-1a+B	0.61	0.008	0.27	0.002	0.44	0.005	0.76	0.018
SCF	0.77	0.054	1.09	0.324	0.46	0.011	1.55	0.053

* Values represent levels of each indicated cytokine in drug-treated, divided by levels in non-drug-treated. Significantly dysregulated cytokines are indicated in grey shading; red for up-regulated and green for down-regulated. Fourteen other cytokines either were not significantly dysregulated, or levels were too low for confident assignment, and are excluded from this table. Values determined from duplicate biologic replicates.
